# Microcomputed tomographic comparison of the bony structure of the canine and feline carpus

**DOI:** 10.1186/s13028-026-00871-8

**Published:** 2026-06-15

**Authors:** Leo Goldstein, Anja-Christina Waselau, Yury Zablotski, Andrea Meyer-Lindenberg

**Affiliations:** 1 Clinic for Small Animal Surgery and Reproduction, LMU, Veterinärstraße 13, Munich, Germany; 2 Clinic for Small Animal Medicine, LMU, Veterinärstraße 13, Munich, Germany

**Keywords:** Bone parameters, Carpus, Cat, Dog, Trabecular architecture

## Abstract

**Background:**

Feline carpal joint injuries pose a significant clinical challenge due to their complex anatomy and the limited evidence available to guide treatment decisions. A detailed understanding of the osseous microarchitecture is essential for improving surgical planning and therapeutic outcomes. A total of 80 carpal joints from 20 cats and 20 toy breed dogs were examined and compared using microcomputed tomography. The parameters bone volume (BV/TV), bone surface (BS/BV), trabecular thickness (Tb.Th), number of trabeculae (Tb.N), trabecular spacing (Tb.Sp), degree of anisotropy (DA) and connectivity density (Conn. D) were measured and compared. To classify the cortical structure, a three-staged scoring system was applied.

**Results:**

All carpal bones exhibited a cancellous structure. Overall, cats had fewer but thicker trabeculae than the toy-dogs in the analysed areas of the carpus, which resulted in a greater trabecular distance in cats. Cats also had a higher bone volume (BV/TV) and thicker cortex.

**Conclusions:**

There were significant differences in structure between dogs and cats which may be important when assessing carpal bone injuries.

## Background

The carpal joint of carnivores consists of seven carpal bones and a large number of ligamentous structures [1]. This complexity which on the one hand allows variable movements, also offers various number of potential pathologies for injuries, which are often the result of direct trauma and usually consist of a combination of bony and ligamentous damage [2]. Depending on their extent, the therapeutic approach to these injuries is temporary immobilisation, ligament repair or replacement or, in severe cases, fusion of the joint by means of partial or total arthrodesis. Surgical techniques will commonly require implant placement into at least one carpal bone [3, 4]. Whether there are differences in the internal bone structure between dogs and cats is usually not given the necessary attention in the current literature. Although the cat is often regarded as a small dog, important anatomical differences and the distinct stresses placed on the feline carpus by behaviours such as climbing and jumping must be taken into account [2, 5, 6]. So far, these anatomical peculiarities of dogs and cats have only been described for the ligamentous structures of the carpus [7, 8]. A detailed study of the architecture of the seven carpal bones is not available in the current literature. In the present study, microcomputed tomographic examinations of feline and canine carpal joints were performed. The primary aim of this study was to evaluate differences in carpal bone structure between dogs and cats of approximately the same weight, to get a direct comparison that is independent of size and weight. A further aim of this study was to create a database on the trabecular and cortical structure of the carpal bones, which could lead to further development or adaptation of implants currently used. Furthermore, this data could also be helpful for the surgical treatment of carpal bone fractures.

## Methods

### Study design

The study included two groups of 20 animals: cats (*n* = 20) and toy dogs (*n* = 20) (Table 1). All carpal joints included were obtained from euthanised or deceased animals. Care was taken to select animals with an estimated age of 8–12 years, to avoid differences caused by osseous remodelling while aging and to ensure a balanced ratio of neutered and unneutered animals. As the exact age was unknown, age was estimated based on dental status and general morphological characteristics. The reported mean age values are based on these estimations. The condition for inclusion in the study was the absence of carpal joint pathologies such as fractures or osteoarthritis [9]. This was verified radiographically in 2 planes, the radiographs were taken before freezing the carpal joints. The radiological examinations were carried out using the Siemens Luminos dRF X-ray unit, Siemens Healthcare AG (Erlangen) with a setting of 50kv and 1.6 mAs.

**Table 1:** Group demographics

Group	Toy breed dogs	Cats
Mean Weight	3.7 ± 1.1 kg	3.5 ± 1.1 kg
Estimated mean age	10.2 ± 2.2 years	8.5 ± 3.2 years
Breeds	Chihuahua (*n* = 5)	ESH (*n* = 17)
	Maltese (*n* = 3)	ELH (*n* = 1)
	Yorkshire-Terrier (*n* = 7)	BSH (*n* = 1)
	Papillon (*n* = 1)	Maine Coon (*n* = 1)
	Miniature-Poodle (*n* = 1)	
	Pomeranian (*n* = 1)	
	Bolonka (*n* = 1)	
	Deer-Pinscher (*n* = 1)	

Before preparation of the joints, the cadavers, which had been stored frozen at −21 °C, were thawed at room temperature for 2 days. The carpal bones were exposed by removing soft tissues, leaving only the ligaments necessary for maintaining anatomical alignment. For better handling, the specimens were separated 2 cm above and below the joint in the antebrachium and metacarpus and stored in 4% formalin until examination in the microcomputed tomograph.

### Micro CT

The joints fixed vertically in the sample container were scanned with 70 kV, 114 µA, 550 ms, a native voxel size of 10 μm and 1000 projections/180° using the microcomputed tomography scanner (µCT-80, Scanco Medical, Zurich, Switzerland). The scan area was chosen to include all seven carpal bones (intermedioradial carpal bone (Cir), ulnar carpal bone (Cu), accessory carpal bone (Ca), first carpal bone (CI), second carpal bone (CII), third carpal bone (CIII) and fourth carpal bone (CIV)). The bones were named according to Nickel, Schummer and Seiferle [1]. The evaluations performed after the scan were carried out using the µCT evaluation programme V6.6 (Scanco Medical, Zurich, Switzerland).

### Analysis of the trabecular bone

**Fig. 1:**
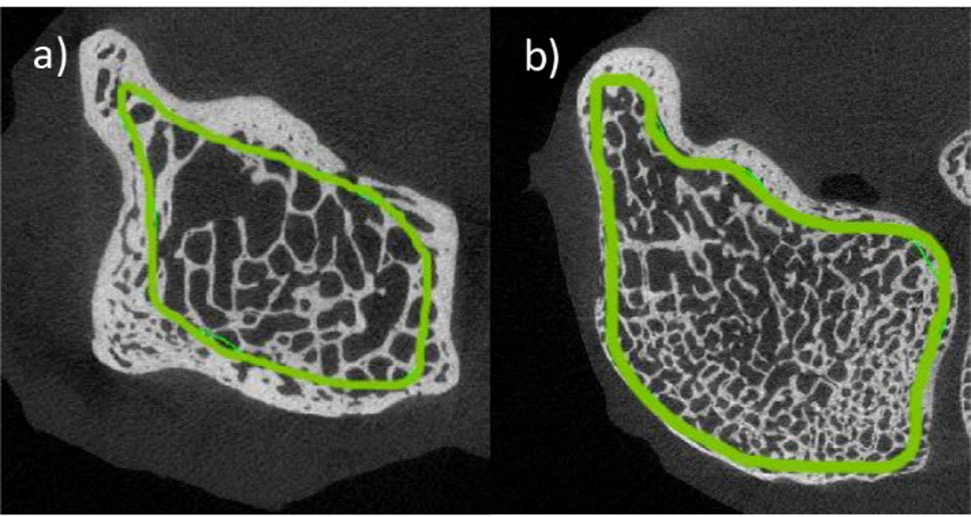
Plotted region of interest (ROI) of the trabecular bone at the thickest part of an intermedioradial carpal bone. **a** European Shorthair Cat. **b** Yorkshire Terrier

To examine the cancellous bone as completely as possible, each scan was reviewed and the most proximal and distal section that still contained cancellous bone was determined. The cancellous bone between these two sections was defined as a region of interest (ROI) for each of the carpal bones (Fig. 1) and subsequently marked by hand in each section [10]. Subsequently, grayscale intensity thresholds for trabecular bone segmentation were determined separately for cats and dogs. Thresholding was applied to the voxel grayscale intensity values of the Micro CT datasets to binarize the images and distinguish trabecular bone from non-bone tissue within the defined ROIs. Species-specific thresholds were used to account for differences in bone size, mineralization, and grayscale distribution between cats and dogs. Threshold selection was performed independently by three experienced reviewers based on visual assessment of representative slices, aiming to achieve optimal delineation of trabecular structures while minimizing inclusion of marrow space and partial volume artifacts. The final threshold applied to each species was defined as the mean of the three independently determined values.

Based on the binarized datasets, the following standard morphometric parameters were calculated according to established definitions for every ROI [11].

- Bone volume fraction (BV/TV): ratio of segmented bone volume (BV) to total volume (TV) within the region of interest, expressed as a percentage.
- Bone surface to volume ratio (BS/BV): total bone surface area (BS) divided by bone volume (BV), indicating surface complexity (mm⁻¹).
- Trabecular number (Tb.N): calculated as the inverse of the mean distance between the mid-axes of the structure (mm⁻¹).
- Trabecular thickness (Tb.Th) and trabecular separation (Tb.Sp): derived using a sphere-fitting algorithm that measures the local thickness of trabeculae and the average distance between them, respectively (mm).
- Connectivity density (Conn.D): measure of the number of trabecular connections per unit volume (mm⁻³).
- Degree of anisotropy (DA): quantifies the preferential orientation of the trabecular structure (dimensionless).”

### Analysis of the cortical bone

**Fig. 2:**
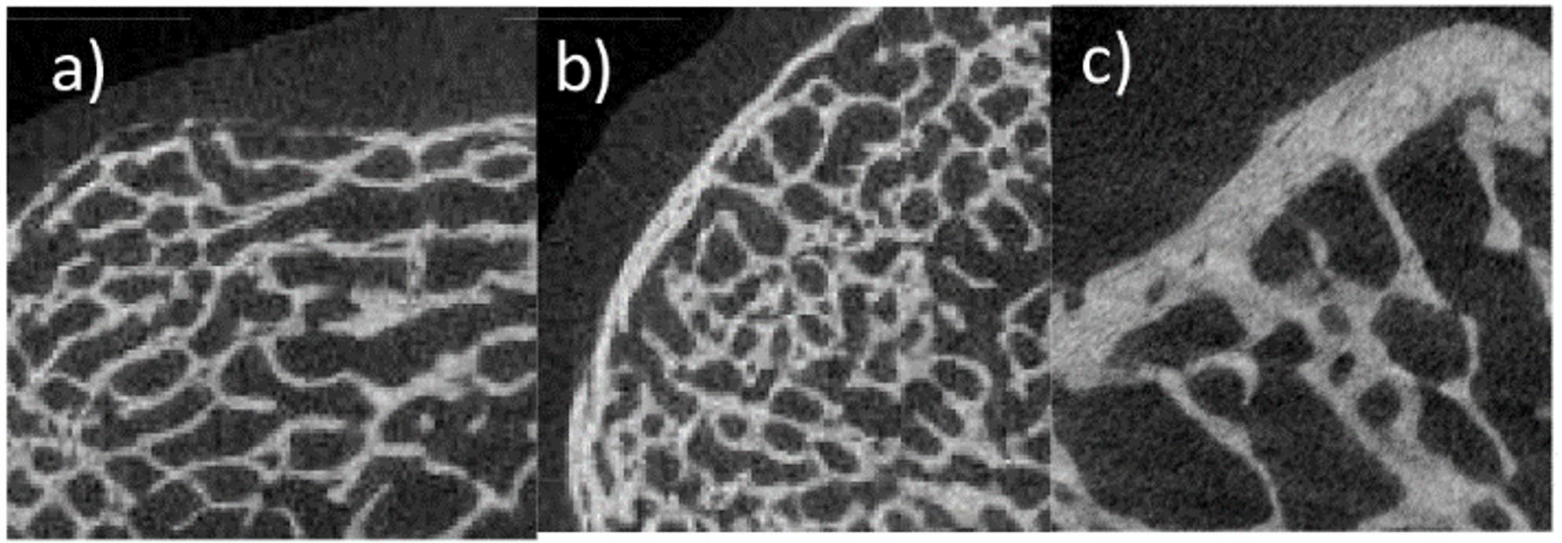
Scoring system for cortical development, as previously published [12]. **a** Score 1: interrupted bone lamella. **b** Score 2: continuous bone lamella. **c** Score 3: normal bone lamella

A previously published evaluation scheme with three scores was used to analyse the cortical portion of the carpal bones [12] (Fig. 2). For the evaluation, each bone was divided into a proximal, middle and distal third and the middle section was used for the assessment, this was carried out by a single reviewer. In addition, the intermedioradial carpal bone, as the largest carpal bone, was further subdivided into three regions (dorsal surface, palmar surface, and the articular surface facing the ulnar carpal bone) to allow a more precise characterization of its cortical structure: The accessory carpal bone was also additionally subdivided into articular and non-articular. The remaining five carpal bones were assessed in their entirety without further subdivision.

### Statistical analysis

Generalised linear models were used to study the differences between toy breeds and cats for every particular carpal bone for the following parameters: BS/BV, BV/TV, Conn.D, cortical score, DA, Tb.N, Tb.Sp and Tb.Th. The following model assumptions were always checked: (1) the normality of residuals was checked by the Shapiro-Wilk normality test, (2) the homogeneity of variances between groups was checked with the Bartlett test, and (3) the heteroscedasticity (constancy of error variance) was checked with the Breusch-Pagan test. In case the assumptions were not satisfied, data were log-transformed and linear models were applied again. All differences between particular groups were assessed after model-fitting by the estimated marginal means (R package-emmeans) with Tukey p-value correction for multiple comparisons. Results with a P-value < 0.05 were considered statistically significant. Data analysis was performed using R 4.2.1 (2022-06-23).

## Results

All included carpal joints could be used for the evaluation. There were no significant differences between the left (*n* = 40) and right (*n* = 40) carpal joints for all seven parameters at all seven localisations and for the formation of the cortical bone (*P* > 0.63).

### Trabecular analysis

All carpal bones analysed in both groups had a trabecular portion. The results of the measurements for the individual carpal bones and groups are listed in Table 2.

**Table 2:** Descriptive statistics

Species	Parameter	Cir	Cu	Ca	CI	CII	CIII	CIV
Toy	BS/BV	20.37 ± 3.73	21.3 ± 3.58	18 ± 3.09	19.86 ± 4.07	17.81 ± 3.83	21.41 ± 3.92	19.64 ± 3.39
Cat	(mm-1)	14.6 ± 2.87	12.33 ± 2.21	13.62 ± 2.31	13.79 ± 2.27	14.49 ± 2.7	13.79 ± 2.28	13.61 ± 2.4
Toy	BV/TV	0.4 ± 0.06	0.4 ± 0.06	0.43 ± 0.07	0.46 ± 0.09	0.5 ± 0.08	0.4 ± 0.07	0.43 ± 0.07
Cat	(%)	0.46 ± 0.09	0.63 ± 0.09	0.48 ± 0.1	0.58 ± 0.12	0.55 ± 0.11	0.57 ± 0.1	0.5 ± 0.08
Toy	Conn.D	58.11 ± 22.12	60.26 ± 20.75	43.81 ± 15.52	56.95 ± 19.14	57.73 ± 23.13	59.29 ± 21.58	60.33 ± 24.24
Cat	(mm-3)	34.38 ± 19.94	32.92 ± 17.99	27.37 ± 15.35	37.16 ± 16.19	37.94 ± 14.57	36.93 ± 15.94	27.01 ± 9.24
Toy	DA	1.27 ± 0.05	1.23 ± 0.04	1.5 ± 0.12	1.44 ± 0.09	1.21 ± 0.06	1.21 ± 0.05	1.35 ± 0.07
Cat		1.22 ± 0.06	1.3 ± 0.17	1.79 ± 0.17	1.42 ± 0.14	1.31 ± 0.16	1.48 ± 0.17	1.35 ± 0.09
Toy	Tb.N	4 ± 0.34	4.18 ± 0.35	3.76 ± 0.4	4.35 ± 0.41	4.3 ± 0.39	4.11 ± 0.38	4.13 ± 0.43
Cat	(mm-1)	3.21 ± 0.34	3.78 ± 0.75	3.18 ± 0.35	3.92 ± 0.48	3.86 ± 0.47	3.82 ± 0.38	3.33 ± 0.26
Toy	Tb.Sp	0.15 ± 0.02	0.13 ± 0.02	0.16 ± 0.04	0.12 ± 0.03	0.12 ± 0.03	0.12 ± 0.03	0.14 ± 0.03
Cat	(mm)	0.17 ± 0.04	0.15 ± 0.03	0.17 ± 0.04	0.14 ± 0.04	0.13 ± 0.04	0.14 ± 0.03	0.15 ± 0.03
Toy	Tb.Th	0.1 ± 0.02	0.1 ± 0.02	0.11 ± 0.02	0.1 ± 0.02	0.12 ± 0.02	0.1 ± 0.02	0.1 ± 0.02
Cat	(mm)	0.14 ± 0.03	0.17 ± 0.03	0.15 ± 0.03	0.15 ± 0.03	0.14 ± 0.03	0.15 ± 0.02	0.15 ± 0.03

Mean values and standard deviations of bone structure parameters for both groups at all evaluated locations

Species: Toy refers to the group of toy breed dogs, and Cat refers to the group of cats

Parameters: BS/BV = bone surface-to-volume ratio; BV/TV = bone volume fraction; Conn.D = connectivity density; DA = degree of anisotropy; Tb.N = trabecular number; Tb.Sp = trabecular separation; Tb.Th = trabecular thickness

Bones: Cir = intermedioradial carpal bone; Cu = ulnar carpal bone; Ca = accessory carpal bone; CI–CIV = first to fourth carpal bones

Cats had a significantly higher bone volume fraction (BV/TV) than toy dogs in all seven carpal bones (*P* < 0.0097). In contrast, the toy dogs showed significantly higher values for the parameter surface volume fraction (BS/BV) on all seven carpal bones (*P* < 0.001). Within cats, the ulnar carpal bone showed the highest bone volume fraction and lowest bone surface to volume ratio of bone values, both statistically significant.

The group of toy dogs had significantly more trabeculae (Tb.N) at all seven localisations than the group of cats (*P* < 0.001) (Fig. 1). The accessory carpal bone had fewer trabeculae than the other 6 carpal bones in both the cats and the toy dogs. The ulnar carpal bone of the cats showed significantly more trabeculae than the other two bones of the proximal joint row (*P* = 0.02).

The trabecular thickness (Tb.Th) was significantly higher in all seven bones in the group of cats (*P* < 0.001) than in the toy dogs. Within the cat group, the ulnar carpal bone had the thickest trabeculae.

The trabecular distance (Tb.Sp) in the cats was significantly higher in all bones than in the toy dogs (Fig. 1), with the accessory carpal bone showing the highest values in both species. The ulnar carpal bone of the cats showed the significantly narrowest distances compared to the other two bones of the proximal joint row (*P* < 0,001).

The connectivity values (Conn.D) of the toy dogs were significantly higher than those of the cats at all seven bones (*P* < 0.001). The accessory carpal bone showed the lowest values in both groups, this finding was not significant.

The geometric degree of anisotropy (DA) was significantly higher at the accessory carpal bone in the cats compared to the toy dogs (*P* < 0.001), and this was also shown with the highest values for the DA (Table 2). For the other six bones, the difference was only slight.

### Cortical analysis

The scoring of the cortical bone revealed that the cat had a significantly higher score (score 3) on all seven carpal bones (*P* < 0.001). It was evenly developed on each bone. In the toy dogs, on the other hand, the cortical bone was only very marginally developed in some cases and sometimes only consisted of an interrupted bone lamella (score 1). This was particularly noticeable on the dorsal surface of the intermedioradial carpal bone and on the carpal bones of the distal joint row. In the proximal joint row, with the exception of the dorsal surface of the intermedioradial carpal bone, the cortical bone was slightly more developed and consisted of a continuous bone lamella (score 2). This difference was observed in all seven carpal bones. The cortical bone was most clearly developed in both groups on the palmar surface of the intermedioradial carpal bone, where it was always normal (score 3). The accessory carpal bone also showed a clearly pronounced cortex at the attachment point of the soft tissue structures (score 3). These findings are observations and not significant differences between bones.

## Discussion

The aim of this study was the microcomputed tomographic visualisation and evaluation of the carpal bones, as there are no comparisons of the exact trabecular and cortical structure in the currently available literature. The differences in carpal bone structure between cats and toy dogs may help guide differences in treatment plans for carpal injuries.

Carpal joint injuries are uncommon in cats, with an incidence of 0.26–0.29% [13, 14], yet surgical treatment carries a relatively high complication rate of 25–35% [2, 15]. This study provides foundational data that may inform the refinement or development of implants and surgical techniques.

Microcomputed tomography (µCT) enables non-invasive three-dimensional imaging of tissue samples, conversely, histomorphometry is based on the analysis of two-dimensional histological sections. As a result, microcomputed tomography provides a more detailed representation of the tissue structure and enables a more precise quantification of morphological parameters, which may be used to predict the mechanical competence of bones [16–19].

Microcomputed tomography, widely used in biomedical research, is also applied in veterinary studies. In dogs, it has examined the medial coronoid process [20–22] and femoral head [23], while in cats it has assessed stifle joint changes after cranial cruciate ligament rupture [24]. Comparative studies of healthy small dogs and cats show that cats have fewer, thicker, and more widely spaced trabeculae, lower connectivity, higher bone volume fraction, and lower surface volume fraction [25, 26]. These patterns, also observed in the carpus in the present study, underscore that cats differ structurally from dogs of similar size and should not be considered merely “small dogs”.

The bone volume fraction (BV/TV) is a measure of the percentage of the analysed sample that is actually bone tissue. High values therefore indicate high bone density and high stability. The BV/TV is therefore one of the primary determinants of the mechanical competence of bone [27, 28]. High BV/TV values also have a good influence on primary implant stability [29]. In the present study, the cats showed significantly higher values for the BV/TV at all seven bones, with the highest values at the ulnar carpal bone.

The BS/BV describes the ratio between bone surface area and bone volume [30]. Low values indicate a lower ratio and therefore a more compact bone structure. In addition, the value is dependent on the number of trabeculae and correlates negatively with the BV/TV [23]. In the present study, the group of cats showed significantly lower values for the BS/BV at all seven bones, again with the lowest values for the ulnar carpal bone.

The number of trabeculae (Tb.N) is an important parameter in describing the bony microarchitecture and correlates strongly with the BV/TV [30]. The number of trabeculae decreases with ageing and the occurrence of microdamage, as is the case in osteoarthritis [31, 32]. In the present study, the toy dogs had significantly more trabeculae on all seven bones than the cats. The lowest values across groups were found for the accessory carpal bone. In the cat group, it was noticeable that the ulnar carpal bone had higher values than the other two bones of the proximal joint row.

Tb.Th stands for the thickness of the trabeculae, the thinner the trabeculae are, the worse this is for stability [24, 27]. In our study, the cats had significantly thicker trabeculae at all locations, with the highest values at the ulnar carpal bone.

The Tb.Sp is a measure of the distance between the trabeculae in the bone tissue and correlates strongly with the stability of the bone [33]. Various diseases lead to a widening of this distance and thus to reduced stability [17, 24, 34]. The cats in this study had significantly wider trabecular distances than the toy dogs, and it was again noticeable that the ulnar carpal bone has much narrower trabecular distances than the other two bones of the proximal row.

The connectivity (Conn.D) of a bone is a measure to quantify the cross-linking of the trabeculae, but it does not allow any conclusions to be drawn about the quality of these connections [30]. Connectivity is negatively correlated with bone stability and should not be considered one of the primary determinants of bone stability [27]. In our own study, the cats showed significantly lower values at all localisations. The lowest values in both groups were localised at the accessory carpal bone.

The degree of anisotropy (DA) is a measure of the spatial orientation of the trabeculae within the bone, it indicates that the properties of bone, like strength or stiffness are direction dependent. Previously, high values have been negatively associated with the mechanical properties and fragility of bone [27, 35]. High DA values indicate that the trabeculae are oriented more in one direction. In this study, the cats showed higher values compared to the toy dogs, with the highest values being found at the accessory carpal bone. This means that the trabeculae of cats are arranged more in one direction, which is consistent with the lower values for Conn.D.

The accessory carpal bone should be considered separately, as it has a different function in the carpus than the other 6 bones. As an apophysis, it is primarily orientated towards tensile loads [36]. It therefore contributes minimally to force transmission along the foreleg and does not require the multidirectional stability of the other carpal bones. Compared to the other carpal bones, it has the fewest trabeculae (Tb.N), which are also less connected to each other (Conn.D), but arranged in one direction (DA) to withstand the tensile loads.

The ulnar carpal bone of cats should also be considered more closely. It has the highest BV/TV, correspondingly the lowest BS/BV, has the narrowest trabecular spacing (Tb.Sp) and the thickest trabeculae (Tb.Th) and also significantly more trabeculae than the other two bones of the proximal joint row, so it appears to be more stable than the other carpal bones. These characteristics are unique to cats and were not seen in the dogs. These features likely reflect the cat’s carpal joint having a much greater range of pronation and supination (≈ 115°) compared to the dog (≈ 50°) [2, 37]. This might lead to more movement and thus a greater load on the ulnar carpal bone and, according to Wollf’s law [38], to functional adaptation of the bone. Because the feline carpus not only acts as a ginglymus, but also allows movement in other planes, this also has an effect on the future treatment of injuries to the carpus [39].

Carpal injuries are uncommon in cats and usually result from falls, known as “feline high-rise syndrome [14]. Surgical management carries a high complication rate [13], making precise implant selection essential [40]. The feline carpus poses additional biomechanical challenges due to its wide range of pronation and supination [39]. In this study, all seven carpal bones showed typical cancellous structure, supporting their potential for implant fixation. The ulnar carpal bone appears particularly stable. Further research is needed to confirm these findings.

The formation of the cortical bone has a major influence on the stability and mechanical properties of bone [41–44], and the holding force of implants is also influenced by it [45]. We suggest that, in cats, the greater cortical thickness may be related to their physiological behaviour (jumping, climbing). In accordance with Wolff’s law [38], increased mechanical loading is known to stimulate cortical bone adaptation and strengthening. Conversely, reduced mechanical stimulation, as may occur in toy breeds could also explain why the cortical bone of toy dogs is sometimes so poorly developed, as these animals are often carried by their owners and thus lack the formative stimulus on the bones. In two previous studies, the authors were also able to show that the cortical bone of the antebrachium and femora in cats is more stable than that of toy breeds [25, 26].

The limitations of this study are primarily due to the partial lack of the animals’ history. Primarily the age should be mentioned here, since for ethical reasons only euthanised or deceased animals were used, whose age was estimated, it can be assumed that these were middle-aged to older. An attempt was made to examine animals between 8 and 12 years of age to minimise age-related influences, as this can have an effect on the number and thickness of trabeculae [31, 46]. Diseases that could lead to a reduction in bony integrity, such as chronic renal insufficiency (CNI) [47] or hyperadrenocorticism (HAC) [48], were excluded as far as the preliminary report allowed. A potential methodological limitation is the use of species-specific thresholds for trabecular bone segmentation. While this approach accounts for biological and grayscale differences between cats and dogs, it may introduce some degree of observer-dependent variability. However, thresholds were determined independently by three experienced reviewers and averaged to reduce subjective bias. Future studies may benefit from automated or standardized thresholding approaches to further enhance reproducibility. Including a group of medium-sized dogs might have provided additional context for interpreting the results; however, our study specifically aimed to compare species at similar body weights to minimize size-related effects.

## Conclusions

Cats and dogs of comparable body weight show distinct differences in the internal microarchitecture of the carpal bones. These species variations may be clinically relevant when selecting fixation methods for carpal injuries and should be considered in surgical planning and biomechanical evaluations.

## Data Availability

The datasets used and/or analysed during the current study are available from the corresponding author on reasonable request.
